# Awake craniotomy for operative treatment of brain gliomas – experience from University Medical Centre Ljubljana

**DOI:** 10.2478/raon-2022-0052

**Published:** 2023-06-21

**Authors:** Tilen Zele, Tomaz Velnar, Blaz Koritnik, Roman Bosnjak, Jasmina Markovic-Bozic

**Affiliations:** Department of Neurosurgery, University Medical Centre Ljubljana, Ljubljana, Slovenia; Department of Neurophysiology, University Medical Centre Ljubljana, Ljubljana, Slovenia; Department of Anaesthesiology and Intensive Care, University Medical Centre Ljubljana, Department of Anesthesiology and Reanimation Faculty of Medicine, University of Ljubljana, Ljubljana, Slovenia

**Keywords:** awake craniotomy, surgery of gliomas, intraoperative neurophysiological testing, primary brain tumours, clinical experiences

## Abstract

**Background:**

Awake craniotomy is a neurosurgical technique that allows neurophysiological testing with patient cooperation during the resection of brain tumour in regional anaesthesia. This allows identification of vital functional (i.e. eloquent) brain areas during surgery and avoidance of their injury. The aim of the study was to present clinical experience with awake craniotomy for the treatment of gliomas at the University Medical Centre Ljubljana from 2015 to 2019.

**Patients and methods:**

Awake craniotomy was considered in patients with a gliomas near or within the language brain areas, in all cases of insular lesions and selected patients with lesions near or within primary motor brain cortex. Each patient was assessed before and after surgery.

**Results:**

During the 5-year period, 24 awake craniotomies were performed (18 male and 6 female patients; average age 41). The patient's cooperation, discomfort and perceived pain assessed during the awake craniotomy were in majority of the cases excellent, slight, and moderate, respectively. After surgery, mild neurological worsening was observed in 13% (3/24) of patients. Gross total resection, in cases of malignant gliomas, was feasible in 60% (6/10) and in cases of low-grade gliomas in 29% (4/14). The surgery did not have important negative impact on functional status or quality of life as assessed by Karnofsky score and Short-Form 36 health survey, respectively (p > 0.05).

**Conclusions:**

The results suggest that awake craniotomy for treatment of gliomas is feasible and safe neurosurgical technique. The proper selection of patients, preoperative preparation with planning, and cooperation of medical team members are necessary for best treatment outcome.

## Introduction

Gliomas are one of the most common primary brain tumours and represent 75% of all malignant primary brain tumours in adults.^1^ The primary treatment consists of surgical resection, followed by radiotherapy and chemotherapy. Despite the modern treatment, gliomas remain incurable lesions.

Glial tumours are classically divided into low-grade gliomas (LGG, WHO grade II) and malignant gliomas (WHO grades III and IV).^1,2^ LGGs are a heterogeneous group, histologically classified into low-grade astrocytoma, oligodendroglioma or mixed oligoastrocytoma. The average age at presentation is 35 years, and they typically occur in the frontal lobes. In 50 to 80% of patients, the first symptom is an epileptic seizure.^2^ On T1-weighted magnetic resonance images (MRI), LGGs appear as hypo- to isointense lesions, whereas on T2-weighted images they are hyperintensive.^2^ Although LGGs are more indolent than their high-grade counterparts, they inevitably progress towards malignancy. This malignant transformation is present in 13 to 86% of cases in LGG recurrences.^2,3^ With treatment, the median overall survival time is about six to eight years.^3,4^ On the other hand, malignant gliomas are more sinister. They are histologically classified into anaplastic astrocytomas (AA, WHO grade III) and glioblastomas (GBM, WHO grade IV).^1^ For AAs, the average age at presentation is 40, and for GBMs 53 years. They typically present with progressive headaches and neurological deficits. On MRI high grade gliomas usually appear as contrast enhancing lesions with central necrosis and surrounding brain oedema. They have a predilection for cerebral hemispheres. With treatment, the median overall survival time for AAs is two to five years and for GBMs less than two years.^5,6^

For patients with gliomas, overall survival was demonstrated to be related to the histological and molecular subtype of the tumour, the patient's age, the presence of neurological deficit, the Karnofsky Performance Status (KPS) score, and the size of the tumour at presentation.^7,8^ In addition, several studies have also clearly demonstrated that the extent of resection (EOR) during surgery has a significant impact on survival.^9,10^ Namely, in cases of LGGs the resection of more than 90% and less than 90% of the tumour resulted in an 8-year overall survival rate of 91% and 60%, respectively.^9^ Similarly, in cases of AAs, gross total resection (GTR) and subtotal resection (STR) resulted in a median survival of 58 and 34 months, respectively.^10^ And in cases of GBM, GTR and STR resulted in a median survival of 13 and 8 months, respectively.^10^

Gliomas are infiltrative lesions and can arise within or near the functionally the most important brain regions, such as language areas, motor cortex, etc. Over extensive resection in those vital (i.e. eloquent) areas, that directly control function, would inevitably result in permanent neurological deficit and significant postoperative morbidity, with a negative impact on the patient's quality of life. In addition, such significant postoperative neurological worsening also independently reduces the overall survival of those patients.^7,8,10,11^ Therefore, the goal of the surgical treatment of gliomas is, in addition to maximal, also safe resection - i.e. removal of as much tumour as possible, without causing neurological deficits (“maximal safe resection”).

To achieve the goal of maximal safe resection, the intraoperative neurophysiological testing and monitoring is mandatory to identify the precise location of individual brain functions and thus eloquent brain areas during surgery. In cases in which neurophysiological techniques cannot adequately assess brain functions under general anaesthesia and the cooperation of the patient during intraoperative testing is needed an awake craniotomy should be used.^12,13^ Awake craniotomy is a neurosurgical technique that allows removal of brain tumour under regional anaesthesia while the patient is awake. It thus allows cooperation of the patient during intraoperative testing and monitoring in order to avoid the injury of eloquent brain areas. For awake craniotomy a variety of anaesthetic methods are used: local anaesthesia (scalp block, infiltration of dura), monitored anaesthesia care (awake-awake-awake technique; scalp block and sedation) or asleep-awake-asleep technique (general anaesthesia and awakening during testing).

In the present article, we presented our protocol, technique and experience with awake craniotomy for glioma surgery at the Department of Neurosurgery, Ljubljana University Medical Centre, from years 2015 to 2019.

## Patients and methods

### Patients

We included all consecutive patients who underwent surgery by awake craniotomy due to gliomas from years 2015 to 2019 at the Department of Neurosurgery of Ljubljana University Medical Centre. The study was approved by the National Medical Ethics Committee of the Republic of Slovenia. All the procedures were performed in accordance with the Declaration of Helsinki. Awake craniotomy was considered in selected patients with the following preoperative morphological images: I) a tumour near or within the language brain areas (Broca's and Wernicke's areas, angular gyrus), II) all cases of insular tumours and III) selected patients with lesions near or within primary motor cortex or corticospinal tract.

Each patient was assessed preoperatively by the anaesthesiologist, neuropsychologist, and neurosurgeon. A collective decision was made whether the patient was suitable for awake craniotomy. The inclusion criteria encompassed a good clinical, physical, and affective condition. The contraindications for awake craniotomy were a non-compliant patient (e.g., due to old age or unfavourable psychosocial factors), the potential for breathing problems during surgery (e.g., known sleep apnoea, significant obesity), and important preoperative dysphasia (i.e., the patient names less than 80% of objects presented at four-second intervals). Informed consent was obtained from all study participants.

### Preo perative planning and evaluation

The preoperative planning consisted of the acquisition and analysis of preoperative morphological and functional images, the selection of surgical approach and trajectory to the lesion, and the planning of the extent of the resection. MRI was performed using a 3-Tesla clinical scanner (Siemens Trio, Siemens). The diagnostic imaging included T1- and T2-weighted sequences. For 3D-imaging, we used a post-contrast T1-weighted 3D-fast spoiled gradient recalled (FSPGR) sequence (a series of 124 images, 1.4 mm thick with a matrix up to 512x512 of 240mm field of view). A fMRI was used to define the primary motor and speech areas in all patients. We used single shot echo planar imaging in a transverse plane (TR 3000/TE 40, a series of 43 images, 3 mm thick with a 64x64 matrix with 200 mm field of view) during which the patient performed a motor task (a self-paced sequential tapping of the thumb against each finger) or a speech task (verbal fluency and verb generation). A general model-based statistical analysis (SPM 12) was used to measure the extent of cortical activation.

To plan the surgical procedure, we used computer assisted 3D-visualization by neuronavigational software (Stealth Station S7 Surgical Navigation System, Medtronic) and 3D-Slicer software (http://www.slicer.org).^14^ The 3D-visualization of the medical images allowed us to perform preoperative 3D-planning, i.e., to interactively present relevant anatomic structures as 3D-objects in virtual space on the computer screen, to define surgical targets, to perform quantitative measurements, and finally to select the most suitable surgical approach or trajectory to the lesion.^15^ Interactive 3D-preoperative planning started with defining the tumour model characteristics (size, volume, extension) and their relationship to the models of the cortical surface and functional data. Based on the available data, we planned the position and size of the trepanation opening, the sites of intraoperative electrophysiological testing, and the preliminary EOR.

### Anae sthetic method

We used awake-awake-awake technique of awake craniotomy - i.e. procedural sedation and analgesia with dexmedetomidine in combination with scalp block. Initially deksmedetomidine infusion (200 μg in 50 ml of 0.9% NaCl; rate 0.2–1.5 4 μg/kg/h) was started to sedate the patient followed by scalp block. Scalp block was performed by local infiltration of the scalp nerves (i.e. n. supratrochlearis, n. supraorbitalis, n. auriculotemporalis, n. zygomaticotemporalis, n. occipitalis minor and major, n. auricularis major) to enabled effective analgesia for skin incision and craniotomy.^16^ For local infiltration of the nerves we used 1–3 ml of the mixture of levobupivacaine (Chirocaine 5 mg/ml solution, Abbvie Pharmacy) and xylocaine with adrenaline (Xylanaest 2% with epinephrin 1: 200000 solution, Kemofarmacija d.d.). Adrenaline causes vasoconstriction and thereby delays absorption of local anaesthetics, increases duration of anaesthesia and prevents local bleeding after skin incision.

In addition, we introduced therapeutic communication with medical hypnosis leaving patients less sedated, more competent during the entire surgical procedure without stress.^17^ Hypnosis session was usually carried out 1 to 3 days before surgery, to gain patients approval and confidence and to teach the patient how to construct an imaginary place where they can feel safe and protected.

Anaesthesia was conducted by two senior anaesthesiologists. The standard intraoperative monitoring was used. All patients had dedicated intravenous and arterial lines, a Foley catheter, and a nasal oxygen catheter. Hypothermia was prevented with use of warming blankets. Cefazolin 2g was used intravenously for perioperative antibiotic prophylaxis.

The patients lay supine with their head rotated approximately 45 degrees to the side. They were encouraged to find the most comfortable position and were generously padded as necessary. The head was fixed in a Mayfield clamp under a scalp block combined with dexmedetomidine. Additional local anaesthesia was applied along the skin incision line and between the dural sheets before the skin and dura incision. Sedation during the procedure was maintained with dexmedetomidine, and painful phases were treated with boluses of remifentanil. Bispectral Index Scale (BIS) was used for sedation monitoring. After the surgical procedure, all patients were transferred to the intensive care unit for overnight observation.

### The operative procedure and neurophysiological testing

Tumour removal was performed in accordance with classical microsurgical techniques. In all cases, microsurgical microscope was employed. During the surgical procedure, frameless neuronavigation, intraoperative neurophysiological testing and augmented malignant tumour visualization with 5-aminolevulinic acid (5-ALA) were used.

For precise intraoperative localization of the tumour, we used neuronavigation with a Stealth Station S7 (Medtronic) navigation station, thus defining the size and position of the trepanation opening and the morphological tumour boundaries on the cortical surface. During tumour removal, neuronavigation was used for approximate depth orientation, and as a help to assess the EOR intraoperatively.

Injury to the eloquent regions was avoided after they had been identified by intraoperative neurophysiological testing. For cortical stimulation, a bipolar electrode was used with biphasic electrical current (50 Hz, 0.5 ms, 4 to 10 mA). For subcortical simulation, a monopolar electrode on the tip of aspirator was used, with a train of five pulses in row (a train of five pulses at 200 Hz, 0.5 ms, from 2 to maximum 20 mA). The patient's speech was monitored by an examiner, who talked to the patient and detected potential speech difficulties. During the stimulation of the cortical surface, the patient named objects on pictures or repeated words after the examiner. When stimulating the speech area, the speech changed or ceased. The stimulation of the primary motor area on the cortical surface resulted in motor evoked potentials (MEPs) detected in the corresponding muscles of the patient's body, and discomfort and/or movements, as reported by the patient. Subcortical stimulation was performed to define the distance from the corticospinal tract in patients where the tumour was in its vicinity. During tumour removal, the subcortical white matter was continuously stimulated with a monopolar electrode, and MEPs were monitored. At the beginning of the resection, the stimulation current was set at 20 mA. When MEPs were detected during the progression of the resection, the stimulation current was gradually reduced. When the MEP response was already induced by a 3–5 mA current, the resection in that direction was stopped, due to the proximity of the corticospinal tract.^18^

In cases where the preoperative diagnostic images showed the characteristics of malignant tumours, 5-ALA was used. The fluorescence of the tumour tissue was observed under the operating microscope (OPMI Pentero 900 Microscope, Carl Zeiss).

### Assessment

The demographic data of the patients were collected before surgery. According to the American Society of Anaesthesiologists, their physical status classification system ASA score was noted and KPS was assessed in all patients. In 12 patients, health-related quality of life (HRQoL) was assessed by 36-Item Short Form (SF-36) health survey.^19,20^

During the surgery, we measured duration of anaesthesia, duration of the operative procedure and duration of the neurophysiological testing. Patient cooperation during surgery was assessed by a neurosurgeon and neurophysiologist and graded on scale from 0 to 10 (modified 11-point numerical rating scale, where 0 represented poor cooperation and 10 excellent cooperation). Patient comfort and pain were assessed by the anaesthesiologist. Pain was assessed by visual analogue scale (VAS) from 0–10 (0 meaning no pain, 10 meaning intolerable pain). Comfort was assessed by modified VAS scale from 0–10 (0 being the least and 10 being the most comfortable). Intraoperative complications were recorded.

After surgery, we monitored postoperative neurological status and noted non-neurological complications. The level of effort needed for the whole procedure, as reported by the patients, was assessed by anaesthesiologist 2–5 days after surgery, by modified VAS scale from 0–10 (0 being the least and 10 being the most difficult). KPS was assessed 1 week after surgery and the patients were asked to describe what was the worst experience during the awake craniotomy. A contrast enhanced brain MRI scan was performed the day following the surgery to assess EOR in all patients. In cases of malignant gliomas (AA, GBM) EOR was assessed on T1 weighted contrast enhanced MRI images. In those cases, gross total resection (GTR) was defined as no residual enhancement on postoperative images, near total resection (NTR) was defined as rim enhancement of the resection cavity, and subtotal resection (STR) was defined as residual nodular enhancement. In cases of LGG, EOR was assessed on T2 and FLAIR weighted postoperative MRI images. In those cases, GTR was defined as no hyperintense signal changes on postoperative images, STR was defined as persistence of hyperintense signal around resection cavity, and biopsy resection (BR) when approximately more than 50% of hyperintense signal remained on postoperative images. Length of hospital stay (LOS) was noted.

Three months after the surgery, the KPS score was assessed in all patients and the HRQoL was assessed by SF-36 health survey in the same 12 patients as before surgery. All patients were asked, if they would be willing to undergo awake craniotomy again if necessary and their answers were noted.

### Statistical analysis

The means or ranges of continuous variables are presented, and categorical data are summarized as counts. The differences between the KPS scores before, after and 3 months after surgery were evaluated using One Way ANOVA. The differences between the SF-36 health survey subscales before and 3 months after surgery were evaluated using Wilcoxon test. A p-value of less than 0.05 was considered statistically significant. Data were analysed by SPSS 13.0 software package (IBM Corp., Armonk, NY, USA).

## Results

### Patients and preoperative data

The demographic and preoperative data are showed in Table 1. During the 5-year study period, we performed awake craniotomies in 24 patients with glial brain tumours. They were 22 to 60 years old. In all cases, the main presenting symptom was epileptic seizure. In addition, in two patients, a mild dysphasia, and in one a disorientation was present. The main comorbidities in patients were well controlled diabetes and arterial hypertension. Four more patients were considered for awake craniotomy but were later found to be unsuitable and underwent surgery under general anaesthesia, one due to anxiety, one due to morbid obesity, one due to asthma and one due to psychosis.

**TABLE 1.** Demographics and preoperative data

**No. of patients**	**24**
Age (years)	41 ± 11
Weight (kg)	81 ± 12
Height (cm)	176 ± 8
Gender (M/F)	18/6
ASA (I/II/III)	9/15/0
First operation/reoperation	22/2
Tumour size (cm^3^)	46 ± 27
Tumour location (side):	
Insular (left/right)	4/3
Frontal Central-PMC (left/right)	2/2
Frontal-Broca area (left/right)	6/1
Temporo-frontal (left/right)	3/0
Temporal-Wernicke area (left/right)	3/0

The results are expressed as mean ± SD or number of patients.

ASA = American Society of Anaesthesiologist; F = female; M = male; PMC = primary motor cortex

**FIGURE 1.**
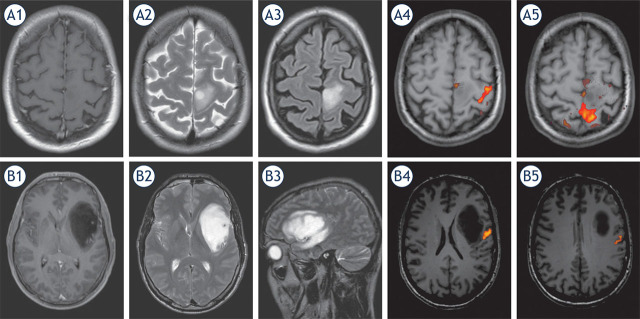
Preoperative magnetic resonance images (MRI) of low-grade glioma located in primary motor cortex **(A)** and anaplastic astrocytoma located in anterior speech area (Broca area) **(B)**. Preoperative imaging included T1 weighted contrast enhanced **(A1, B1)**, T2 weighted **(A2, B2)**, FLAIR **(A3, B3)** and functional magnetic resonance (fMRI); **(A4, A5, B4, B5)** images. The fMRI demonstrated the hand activation area (yellow/red colour) lateral (A4) and leg activation area behind **(A5)** the lesion in primary motor cortex. In second case fMRI demonstrated speech activation areas posterior to the lesion on the left side **(B4, B5).**

The preoperative fMRI was successful in all patients and revealed speech dominance on the left side in all but one case in which it was bilateral. In 4 cases with tumour in frontal-central region (i.e., near or within primary motor region) the fMRI was used also to define primary motor regions (Figure 1).

### Intraoperative and early postoperative results

Intraoperative and early postoperative outcomes are presented in Table 2. The longest surgical procedure lasted 5 hours and 20 minutes. Patient cooperation during surgery was in majority of the cases excellent. Discomfort during surgery reported by the patients was generally described as just a little uneasy and/or just starting to bother. Pain perceived during surgery was regarded as moderate. In two patients, however, we observed significant fatigue during surgery that started about 2.5 hours after the start of the procedure, and it was so intense that further neurophysiologic testing was not feasible. During cortical stimulation, we observed epileptic seizure activity in 8 out of 24 patients (33% of the patients). In one of these patients, further testing was not possible, due to limited compliance after the seizure. Neurophysiological testing by cortical stimulation was therefore successful in identifying the cortical language areas or primary motor cortex in all but three cases. Subcortical stimulation was performed in cases with insular and frontocentral located tumours. The corticospinal tract upon stimulation with a 20 mA current or less was detected in 8 out of 11 tested patients (72% of the patients).

**TABLE 2.** Intraoperative and early postoperative outcomes

**INTRAOPERATIVE DATA**	
Duration of anaesthesia (minutes)	278 ± 47
Duration of procedure (minutes)	215 ± 48
Duration of testing (minutes)	73 ± 26
Comfort score (0- least; 10- most)	8 ± 2 (5–10)
Pain score (0- no pain; 10- intolerable)	4 ± 2 (0–5)
Cooperation score (0- poor; 10- excellent)	10 ± 1 (9–10)
Complications (none/seizure/incomplete testing)	13/8/3

**EARLY POSTOPERATIVE DATA**	
Pain score after procedure (0- no pain; 10- intolerable)	2 ± 1 (0–3)
Pain score ICU (0- no pain; 10- intolerable)	2 ± 1 (0–3)
Level of effort (0- least; 10- most difficult)	3 ± 2 (0–6)
Non-neurological complications	
Wound infection	1
Pulmonary embolism	1
Nausea	2
Pain	2
Neurological complications	
Walking disability	1
POCD and dysphasia	1
Mild hemiparesis, POCD, seizures	1
Tumour histology	
LGG	14
AA	7
GBM	3
Extent of resection	
LGG (GTR/STR/BR)	4/7/3
AA or GBM (GTR/NTR/STR)	6/3/1
Length of stay (days)	6 ± 5

The results are expressed as mean ± SD (range in brackets) or number of patients.

AA = anaplastic astrocytoma; BR = biopsy resection; GBM = glioblastoma; GTR = gross total resection; ICU = intensive care unit; LGG = low grade glioma; NTR = near total resection; POCD = postoperative cognitive decline; STR = subtotal resection

**FIGURE 2.**
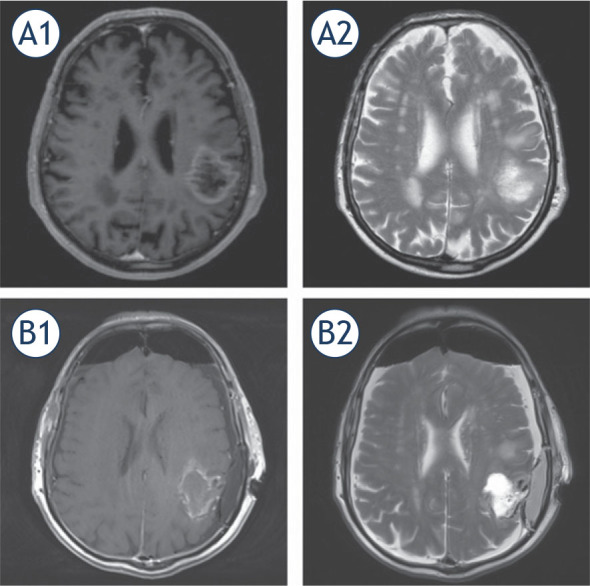
Preoperative **(A)** and postoperative **(B)** T1 weighted contrast enhanced (A1, B1) and T2 weighted (A2, B2) magnetic resonance images (MRI) of glioblastoma located in supramarginal gyrus near Wernicke area. Preoperative images **(A)** demonstrate rim enhancing central necrotic oval lesion surrounded by oedema. Postoperative images **(B)** demonstrate post-resection cavity filled with partially haemorrhagic fluid and mild irregular contrast enhancing of resection edge – i.e. near total resection.

After the surgery, the neurological worsening was observed in 3 out of 24 patients (13% of the patients) and the other postoperative complications were present in 6 out of 24 patients (25% of the patients) (Table 2). Postoperative pain was mild in almost all cases. Level of the effort during surgery experienced by the patients was assessed and reported to be mild to moderate. When asked “what was the worst experience during the awake craniotomy”, 15 patients said “nothing”, four patients said, “bone cutting”, two patients said, “Mayfield clamp placement”, and the remaining three patients said, “scalp block”, “craniofix bone flap fixation” or “waiting for the end of surgery”. GTR was achieved in 29% (4 out of 14 cases) in cases of LGG, and in 60% (6 out of 10 cases) in cases of GBM and AA (Figure 2).

All patents were discharged home. The time of postoperative hospital stay was from 3 to 9 days for most of the patients, and 23 days for one patient.

### Late postoperative results

**FIGURE 3.**
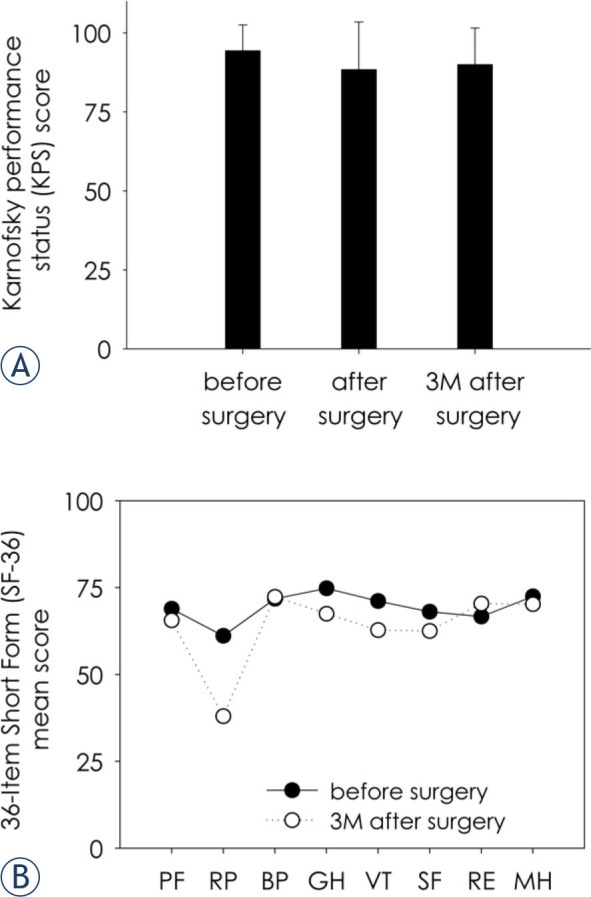
The Karnowski Performance Scale (KPS) scores **(A)** and 36-Item Short Form (SF-36) health survey subscale scores **(B)** before surgery, after surgery and 3 months after surgery. The differences between the KPS scores and SF-36 subscale scores before and after surgery were not statistically significantly different (p > 0.05; n = 24 for KPS; n = 12 for SF-36). BP = bodily pain; GH = general health; PF = physical function; MH = mental health; RE = role limitations due to emotional problems; RP – role limitations due to physical problems; SF = social functioning; VT = vitality; 3M = three months

Three months after surgery all patients were under postoperative oncological treatment with radiotherapy and/or chemotherapy. All patients were still on a sick leave, and none yet returned to work. The average KPS scores after surgery and 3 months after surgery were slightly lower and are shown in Figure 3A. However, the differences between the KPS scores before, after and 3 months after surgery were not significantly different (p = 0.14). Patient HRQoL was assessed by SF-36 health survey before surgery and 3 months after surgery, the results for each SF-36 subscale are shown in Figure 3B. We found some increase in patient perceived problems with work or other daily activities because of physical health (i.e., “role limitations due to physical problems” SF-36 subscale) three months after surgery in comparison to before surgery, however, the difference was not statistically significant (p = 0.07). The scores in other SF-36 subscales (i.e., physical functioning, bodily pain, general health, vitality, social functioning, role limitations due to emotional problems, mental health) were very similar before and after surgery, and not significantly different (p > 0.1). Therefore, the surgery did not have important negative impact on functional status or quality of life as assessed by KPS and SF-36 health survey, respectively. Three months after surgery, the patients were asked, “if they would be willing to undergo awake craniotomy again if needed”. One patient answered “no”, one answered, “only if absolutely necessary” and the other 22 patients answered “yes”.

## Discussion

At our institution, awake craniotomy for surgical treatment of gliomas was performed in 24 patients in the last 5 years. Our experiences with awake craniotomy are good and suggest that awake craniotomy can be safely performed with low risk of complications or failure rate.

The goal of an awake craniotomy is to provide the surgeon with optimal functional monitoring during the removal of as much of the tumour as possible and thus avoiding damage to critical brain structures. Awake craniotomy is mandatory in patients where neurophysiological testing and monitoring cannot adequately assess function under general anaesthesia, and when the cooperation of the patient during testing is needed. The functions typically monitored during awake craniotomy are mainly higher mental functions, especially speech. Such monitoring allows safer tumour removal, resulting in improved treatment outcome, even for tumours in eloquent brain regions.^12,13,18,21,22^

On the other hand, awake craniotomy is a technically demanding procedure, with more potential intraoperative complications in comparison to the same procedure performed under general anaesthesia.^21^ Namely, patient intolerance or non-cooperation can result in too early termination of the surgery, and incomplete or suboptimal resection.^21^ Therefore, it is very important to properly select patients for the procedure. In our series, all the patients performed well, and the selection protocol for the patients was shown to be good. During preoperative evaluation, four potential candidates were found to be unsuitable for awake craniotomy. In addition to obesity, potential breathing problems and dysphasia, also patient immaturity, hypertension and alcohol abuse are also some of the risk factors for sedation failure, which may worsen the surgery and postoperative recovery.^23^ Anxiety and other psychological problems may also negatively influence the patient's cooperation.^24^ The selection of patients should therefore be individualized, and the decision-making process should involve all team members.

A variety of anaesthetic methods are used for awake intracranial procedures. In our institution, we combine scalp block and procedural sedation and analgesia with dexmedetomidine – i.e. awake-awake-awake protocol. This technique proved to be very safe in experienced hands, allows more control with the patient, the anaesthesiologist is by the patients side all the time and can react promptly if something goes wrong, and it usually results in better patient cooperation.^25,26^ All patients are well-educated regarding all the steps of the surgical procedure to facilitate the intraoperative testing and to decrease their anxiety. In our, and also other studies, the most painful and unpleasant part of the procedure, as reported by the patients, is the placement of the cranial fixation device and trepanation.^27,28^ It is important to note that because of inadequate analgesia during cranial fixation, or later due to positioning, the surgical outcome may be compromised.^29^ The patient's communication with the surgical team and addressing the patient's discomfort are therefore crucial. In the case of the awake-awake-awake protocol, as used in our cases, the duration of the surgery proved important. The cooperation of two of our patients became inadequate after approximately 2.5 hours from the beginning of the surgery. To overcome this problem, we recommend early functional testing and analysis of the functions for which patent's cooperation is needed. In addition, we recommend good preoperative planning and preparation to reduce operation time.

In our study, the patient's perception of awake craniotomy was generally good. Hypnosis was well accepted in our study. Also, it was previously shown that it prolongs the time of patient cooperation during the neurophysiological testing.^30^ The main challenges for patients undergoing awake craniotomies include anxiety and fears, terrifying noises and surroundings, immobility, loss of control, the feeling of helplessness and being left alone. In such situations, psychological support might be very helpful and motivates patients to have a sense of control by active participation during surgery instead of being lost in anxiety.^31,32^

The rate of occurrence of epileptic seizures during surgery in our study of 33% was higher compared to other reports, which reported a seizure rate of from 15 to 16.7%.^21,28^ At the beginning of the operation, cortical stimulation, especially when used in excess during neuromonitoring, may sometimes provoke epileptic seizures.^21,28^ Reported treatment was the iced Ringer solution, which was also successfully used in our patients to stop the seizure. We believe that the higher rate in our series could be attributable to more extensive testing in selected patients. In this regard, it was observed that less stimulation is generally used with increased experience.^28^

The neurological worsening observed postoperatively in 13% of our patients is comparable to the meta-analysis data, which report rates of about 30% for early and 7% for late deficits of all severities after intraoperative stimulation mapping.^33^ GTR in our study was higher in cases of GBM or AA in comparison to LGG (29% vs. 60%). The comparable GTR rates are also reported in the literature for awake craniotomy glioma surgery.^34,35^ In this regard, it is important to note, that although 5-ALA and neuronavigation help to delineate the borders of the tumour tissue, the final edge of the resection is defined by the functional borders, as defined by intraoperative neurophysiological testing. Consequently, in several cases of awake craniotomy total resection of the glioma cannot be achieved due to tumour infiltration of eloquent regions. Based on intraoperative neurophysiological testing, we believe that further resection in our patients could result in significant postoperative functional deficit.

One of the goals of surgical treatment of gliomas is, in addition to maximal resection, preservation of the neurocognitive profile and quality of life.^19,20^ Namely, gliomas are currently surgically incurable lesions and potential postoperative neurological deficits further reduce survival time and quality of life of those patients. In our study, the HRQoL assessed three months after surgery was not significantly impaired. We did however observe noticeable patient reported problems with work and daily activities three months after surgery. The later could be related to ongoing postoperative oncological treatment. Also, none of the patients at 3 months after the surgery was yet allowed to return to work, which probably also influenced their perception and the results.

To conclude, our experience suggests that awake craniotomy using our protocol is a feasible and safe surgical procedure. To achieve the best treatment outcome, proper selection of the patients, preoperative preparation with planning, and cooperation of the medical team are necessary.
